# Plasmonic Color Filter Array with High Color Purity for CMOS Image Sensors

**DOI:** 10.3390/s19081750

**Published:** 2019-04-12

**Authors:** Atsutaka Miyamichi, Atsushi Ono, Keiichiro Kagawa, Keita Yasutomi, Shoji Kawahito

**Affiliations:** 1 Graduate School of Science and Technology, Shizuoka University, 3-5-1 Johoku, Naka-ku, Hamamatsu 432-8561, Japan; miyamichi.atsutaka.15@shizuoka.ac.jp (A.M.); kagawa@idl.rie.shizuoka.ac.jp (K.K.); kyasu@idl.rie.shizuoka.ac.jp (K.Y.); kawahito@idl.rie.shizuoka.ac.jp (S.K.); 2 Research Institute of Electronics, Shizuoka University, 3-5-1 Johoku, Naka-ku, Hamamatsu 432-8011, Japan

**Keywords:** surface plasmon, color filter, multiband, image sensors, metallic thin film

## Abstract

We demonstrate the multiband color filtering of a standard RGB color and a complementary CMY color by a plasmonic color filter, composed of concentric corrugated metallic thin film rings. The surface plasmon resonance is excited by the periodic corrugation, and the coupled light is transmitted through the central subwavelength aperture. Color selectivity is achieved not only in the visible but also in the near-infrared (NIR) region. Therefore, simultaneous imaging with visible and NIR can be realized by the integration of plasmonic color filters with sensors. We investigate the angle of incidence dependence of the transmission color selectivity and the color purity of the fabricated plasmonic color filter array.

## 1. Introduction

The multiband imaging technique, which integrates the advantage of conventional imaging and spectroscopy, has been proposed as an approach to simultaneously acquire spatial and spectral information in various application fields, such as product evaluation, food inspection, biomedicine, and aerospace. The multiband imaging system, composed of a monochrome camera and a rotating filter wheel holding a series of optical filters, has been studied extensively [1,2,3]. Although such systems are easily constructed, a multiband camera implementing a tunable filter with a spectral transmission that can be controlled electronically has been developed in order to eliminate the mechanical rotation of the filter turret [4,5]. These imaging systems in which a filter is externally attached to the image sensor can capture light with a high sensitivity and color purity because of the high utilization efficiency of light but are bulky, occupying a large volume. For downsizing, multispectral filter arrays with different colored filters at each pixel have been studied for multispectral imaging [6,7,8]. Multiband images can be reconstructed using the demasking method by acquiring one channel at each pixel as well as the Bayer color filter array in a conventional RGB camera [9]. This filter array-based imaging system is compact and highly compatible with real-time image capture because a multiband image can be obtained in one exposure; however, the number of bands is limited, and the light utilization efficiency is low because of a reduced transmittance.

The color filters widely used in the current image sensors are polymer-based materials with dyes or pigments. While the polymer-based color filters are characterized by a high transmittance and vivid color purity, they are costly and impractical for multiband imaging applications because of the requirement of several lithography steps for each color. Further, the spectral properties of the filters are not easy to tune because they depend upon the properties of the polymer materials. Further, the polymer layer cannot be made thinner than a few hundred nanometers without impacting the filtering efficiency owing to the low absorption coefficients of dye and pigment materials. Thus, because of the thickness of color filters, the deterioration of color reconstruction by a spatial optical crosstalk between adjacent pixels occurs for a light incident at a large angle. As an alternative technique to conventional color filtering, a new color filtering approach using the surface plasmon resonance (SPR) excited on metallic periodic nanostructures thinner than a few hundred nanometers have been actively studied [10,11,12,13,14,15,16,17,18,19]. Ebbesen demonstrated that the light of a specific wavelength is transmitted from nanohole arrays periodically patterned in a metal thin film [20]. The wavelength of light desired to be transmitted is accommodated by changing the period of the array. The optical transmission of plasmonic hole arrays occurs due to the coupling of the diffracted light and the surface plasmons by the subwavelength aperture array, which acts as a diffraction grating. The dispersion curve of the diffracted light shifts depending on the period; the surface plasmons are excited on the surface of the metal thin film when the wavenumbers are matched. The excited surface plasmons are diffracted by the periodic structure of the same wavenumber and propagate through the aperture. The incident light that does not couple with surface plasmons is reflected by the bulk metal; thus, a wavelength selectivity occurs at the periodical nanostructure. The parallel component of the incident wave vector $k_{x}$ is described by Equation (1),

(1) $$k_{x}=k_{0}sin\theta +mK$$

where $k_{0}$ is the magnitude of the incident wave vector in free space, θ is the angle of incidence (AOI) of light, *m* is an integer corresponding to the diffraction order, and *K* is the grating vector. In the case that the incident wave vector and the wave vector of the surface plasmon $k_{spp}$ match, a surface plasmon is excited. The wave vector $k_{spp}$ is determined by Equation (2),

(2) $$k_{spp}=\left(\frac{\omega }{c}\right)\sqrt{\frac{\epsilon_{d}\epsilon_{m}}{\epsilon_{d}+\epsilon_{m}}}$$

where $\epsilon_{d}$ is the permittivity of the dielectric material and $\epsilon_{m}$ is the permittivity of the metal. An optical filter that transmits light of a desired wavelength can be easily designed by tuning the period; thus, multiband imaging using plasmonic structures tuned to each band is anticipated.

## 2. Proposed Multiband Plasmonic Color Filter

**Figure 1:**
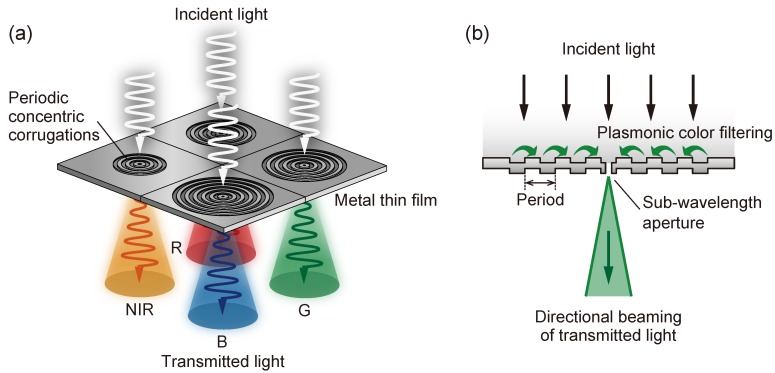
(**a**) A schematic of the proposed multiband plasmonic color filter. (**b**) The mechanism of selected wavelength transmission by surface plasmon coupling.

We proposed a multiband plasmonic color filter transmitting from the visible to the near-infrared (NIR) regions and demonstrated a multiband wavelength selectivity [21]. Figure 1a shows a schematic of the proposed multiband plasmonic color filters. An image sensor that discriminates the visible (RGB) light and NIR light simultaneously can be realized by integrating the proposed plasmonic color filter to 2×2 pixels as a single unit. We applied a single subwavelength aperture surrounded by periodic concentric corrugations for the plasmonic structure. The selected wavelength propagates as a beam from the aperture, as first proposed by Lezec et al. and reported in 2002—the so-called “bull’s eye” [22]. The plasmonic hole arrays in previously reported studies have demonstrated an optical transmission with vibrant colors that depends on the periodicity of nanoholes. However, the spectral width of the transmitted light is approximately 150 nm, whereas an increased wavelength selectivity resulting in a narrower transmitted waveband is required for multiband imaging constituted by several tens of wavebands. The proposed structures have a smaller dispersion of the resonant wavelength than the nanohole arrays. The proposed plasmonic color filter provides a higher wavelength selectivity and narrower spectral bands than the nanohole array filter, predominant in multiband imaging. Figure 1b shows the cross-sectional view of the proposed plasmonic color filter. A particular wavelength of the incident light couples with the surface plasmons on the input side of the structure. The light of the coupled wavelength propagates and concentrates at the central aperture that disturbs the periodicity of the corrugation.

The directionall beaming of the transmitted light is caused by a plasmon coupling mechanism on the emission side of the aperture similar to that of the incident side. This directional beaming property suppresses the undesirable optical crosstalk of adjacent pixels, leading toaan improved color reconstruction. This “bull’s eye” structure concentrates the light of the wavelength for which the filter has been designed and forms a localized electric field enhancement around the central aperture, which acts as a plasmonic antenna, leading to the improvement of the external quantum efficiency of the nano-photodiode [23]. In this work, we report a multiband color transmission from the visible to the NIR achieved by tuning the period of the concentric corrugation.

**Figure 2:**
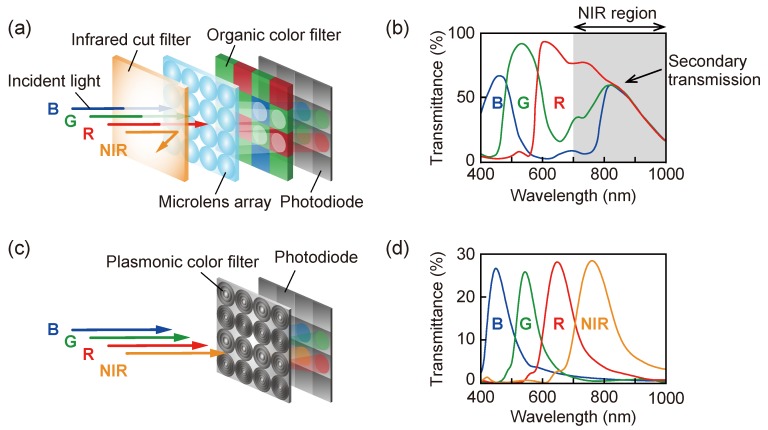
(**a**) The general pixel configuration by using organic color filters and (**b**) the typical RGB transmission spectra of organic color filters. (**c**) A schematic of the proposed configuration and (**d**) the corresponding simulated RGB–near-infrared (NIR) transmission spectra showing the selectivity of the spectra.

In the conventional polymer-based color filter, it is difficult to simultaneously discriminate between visible light and NIR light. Figure 2a,b shows a schematic of the general configuration of pixels and the corresponding transmission spectra.

Although the organic color filter used for the current complementary metal-oxide semiconductor (CMOS) image sensors has a high transmittance, secondary optical transmission occurs in the NIR, a sensitive spectral region of Si. This secondary transmission causes a deterioration of the color reconstruction because NIR light is incorrectly detected at the RGB pixels and the original RGB signals are diluted. In order to suppress any signal resulting from NIR light, the sensitivity of the image sensor is restricted to the visible by an infrared cut filter. Consequently, it is difficult to simultaneously detect and discriminate between the visible and NIR light. In previous research, simultaneous imaging systems without an infrared cut filter used the difference processing of the RGB image, and NIR images have been studied by assigning an NIR bandpass filter to one pixel [24,25,26]. Figure 2c,d shows a schematic of the pixel configuration of the proposed plasmonic color filter and the transmission spectra corresponding to R, G, B, and NIR simulated by using the finite-difference-time-domain (FDTD) algorithm. Plasmonic color filters that transmit the desired wavelength as a bandpass corresponding to the corrugation period make it possible to eliminate the infrared cut filter. Therefore, both visible and NIR light can be imaged simultaneously by the integration of a suitable plasmonic filter array, with filters corresponding to R, G, B, and NIR with the CMOS image sensor. The proposed plasmonic color filter facilitates the simultaneous discrimination of visible and NIR imaging for various applications, such as vehicle mounted cameras, day/night security cameras, and biological tissue engineering.

## 3. Numerical Simulation; AOI Dependence of Transmission Spectra

**Figure 3:**
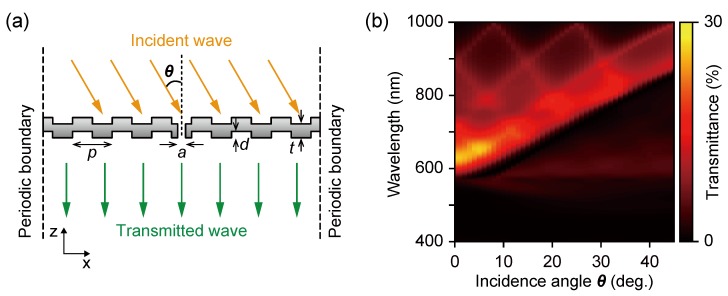
(**a**) The simulation model for the angle of incidence (AOI) dependence of the transmission spectrum. (**b**) The simulated transmittance spectra of AOI dependence for values of *p* = 500 nm, *d* = 80 nm, *t* = 180 nm, and *a* = 90 nm.

We analyzed the AOI dependence of the transmission spectra in order to investigate the effective cone angle acting as a color filter. Figure 3a shows a schematic of the simulation model as a one-dimensional periodic corrugated silver thin film. The corrugations are periodically distributed and symmetrically located with respect to the central aperture. The transmission spectrum as a function of AOI was simulated by a rigorous coupled wave analysis (RCWA), performed using commercial software (DiffractMOD, Synopsys Inc.). The filter model is defined by the following structural parameters: *p* (corrugation period), *d* (groove depth), *t* (film thickness), and *a* (aperture diameter). The simulation domain of the *x*-direction was defined as seven corrugation periods. The incident light was defined as a plane wave at an AOI θ with *p*-polarization. Incident *p*-polarized light excites surface plasmons because the electric field has a component perpendicular to the corrugation direction. The transmitted power of each individual diffraction order is calculated and summed as the transmittance. Figure 3b shows the AOI dependence of the transmission spectrum for a plasmonic color filter with *p* = 500 nm, *d* = 80 nm, *t* = 180 nm, and *a* = 90 nm. As shown in the figure, the surface plasmons excited on the silver surface are diffracted by the periodic corrugations and are indicated as a dispersion curve of the surface plasmons coupled to the propagating light for each diffraction order.

A transmission peak wavelength of 650 nm was indicated at normal incidence, θ = 0°, shifting to longer wavelengths with an increasing AOI. In the AOI dependence from θ = 0° to θ = 45°, the peak wavelength of the transmission spectrum was constant up to the cone-angle of approximately 10°.

## 4. Fabrication Process

**Figure 4:**
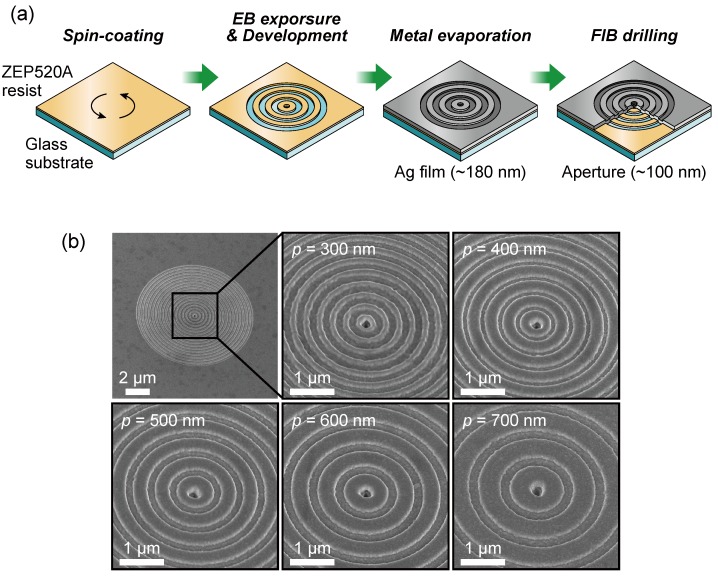
(**a**) The fabrication process for the periodic concentric corrugated plasmonic film. (**b**) The tilt SEM images of the fabricated structures with a corrugation period of 300–700 nm.

The proposed plasmonic color filter was fabricated on a glass substrate by using a standard liftoff process, performed by electron beam lithography (EBL), vacuum evaporation, and a focused ion beam (FIB). Figure 4a shows a schematic of the key fabrication process steps for the proposed plasmonic color filters. The first step is spin-coating an electron beam resist on a clean glass substrate. A ZEP520A (Zeon Corporation, Chiyoda, Japan) positive-type electron beam resist diluted with a ZEP-A organic solvent, diluted at 1:1.6 by weight, was spin-coated at a rotation speed of 4500 rpm and prebaked at 180 °C for 2 min. An Espacer 300Z (Showa Denko K.K., Minato, Japan) conductive polymer layer was spin-coated over the resist layer to prevent charge-up. The resist was exposed to the electron beam using an EBL system (ELS-7700K, ELIONIX INC., Hachioji, Japan) at an acceleration voltage of 80 kV and current of 50 pA to form a nanoscaled periodic concentric pattern. Then, the conductive polymer was removed by ultrapure water, and the exposed substrate was developed by dipping it in o-Xylene. The sample was rinsed with isopropyl alcohol and then post-baked at 120 °C for 2 min. After the development process, a silver thin film 180-nm thick was deposited at a rate of 0.2 nm/s by vacuum evaporation. By evaporating the silver film onto the patterned resist, a silver nanostructured pattern is also formed on the surface. Thus, the nanoscaled periodic concentric corrugation was fabricated on both silver surfaces. The groove depth *d* of the fabricated structure was 90 nm owing to the thickness of the resist layer spin-coated on the glass substrate. Finally, a subwavelength aperture of approximately 100 nm in diameter was drilled at the center of concentric corrugation by using a FIB system (JIB-4500, JEOL Ltd., Akishima, Japan) at an acceleration voltage of 30 kV and a beam current of 10 pA.

Figure 4b shows 30° tilted SEM images of the fabricated plasmonic color filters with a corrugation period of 300–700 nm. The number of grooves was adjusted for each corrugation period so that the outer diameter of the filter size was approximately 10 μm. It was verified by observation that the periodic corrugations and subwavelength aperture were fabricated in the 180-nm silver thin film. In this study, the transmitted light distribution and transmission spectrum of the fabricated structures were measured by using a xenon white light source and a transmission light microscope.

## 5. Results and Discussion

### 5.1. Transmission Color Purity of Plasmonic Color Filters with Periodic Concentric Corrugations

**Figure 5:**
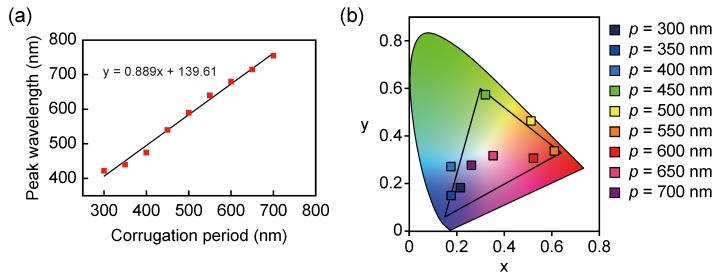
(**a**) The peak transmitted wavelength as a function of the corrugation pitch in 50-nm steps and (**b**) the CIE1931 XY chromaticity diagram of the measured transmission spectra.

We have demonstrated the transmission spectra that exhibits the wavelength selectivity of the visible and near-infrared range [21]. In this section, we evaluated the color purity of the transmitted light in the fabricated structure. Figure 5a is a plot of the transmission peak wavelength for the various corrugation periods of the fabricated plasmonic color filter (*p* = 300–700 nm in 50-nm steps). The transmission peak wavelength was linearly shifted to longer wavelengths from 420 nm to 755 nm with increasing corrugation period. This linear dependence of the filtering wavelength on the corrugation period is practical for the design of color filters. In addition, the transmission peaks observed from the visible to the NIR region are appropriate for the multiband imaging from visible to NIR. The silver of the filter material has low optical absorption losses owing to its complex permittivity. Therefore, it is possible to extend the filtering wavelength up to the 1-μm band gap of Si by increasing the corrugation period. Figure 5b shows a CIE1931 XY chromaticity diagram of the measured transmission spectra plotted as XY color coordinates. As shown in the chromaticity diagram, the transmitted light shifts from blue to red as the corrugation period increases. The corrugation periods corresponding to the three primary colors of red (R), green (G), and blue (B) are *p* = 550 nm, 450 nm, and 350 nm, respectively. These XY color coordinates are almost coincident with the vertices of the black triangle that bounds the standard RGB (sRGB) color space. Since the corrugation period of the filter was fabricated in 50-nm steps, not only were the primary colors of RGB but also the complementary colors of cyan (C), Magenta (M), and Yellow (Y) were obtained. In particular, the color coordinates of the transmitted light corresponding to *p* = 400 nm (C) and 500 nm (Y) exceed the sRGB color gamut. The fabricated plasmonic color filter exhibited a high color reproducibility in the visible region. The transmission peaks corresponding to the color coordinates of the color filter with *p* = 650 nm and 700 nm were in the NIR region and are shown near the neutral point exhibiting saturated colors because the transmitted light was scarcely observed in the visible range.

### 5.2. Dependence of Transmitted Light on Number of Grooves

**Figure 6:**
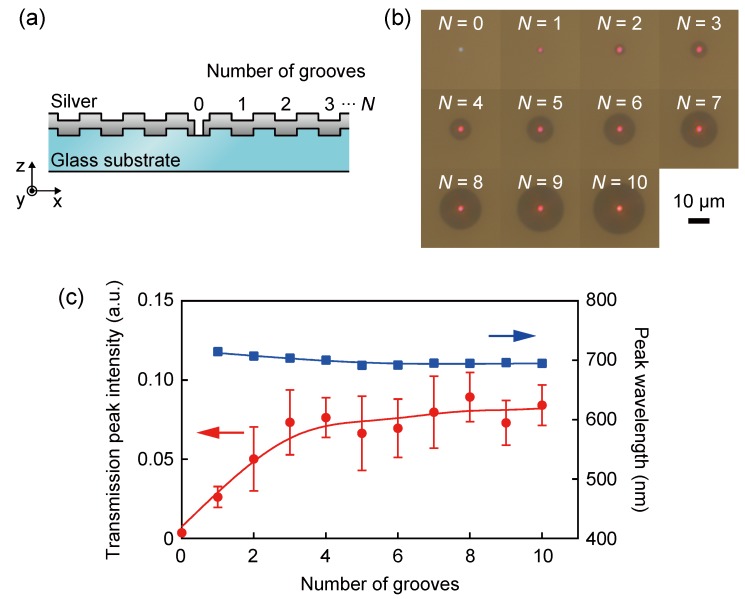
(**a**) A cross-sectional schematic of the corrugated plasmonic film. (**b**) Optical micrographs of the groove number dependence of transmitted power for *N* = 0 to 10. (**c**) The transmission peak intensity and peak wavelength dependence on groove number for *N* = 0 to 10.

We defined the central aperture and the number of annular grooves *N* in the periodic corrugated silver thin film as shown in the cross-sectional view of Figure 6a. Figure 6b shows optical micrographs of the transmitted light from the fabricated plasmonic color filter with *p* = 600 nm when the number of annular grooves is increased from *N* = 0 to *N* = 10. In a comparison of the transmitted light distribution between *N* = 0 and *N* = 10, it was clearly observed that the structure with *N* = 1 transmits the red color light, which was not observed for the structure without the annular grooves. Therefore, even if there is only one annular groove surrounding the aperture, the annular grooves contribute to the color filtering from the subwavelength aperture as a periodic corrugation inducing surface plasmon resonance. Figure 6c shows the measured transmission peak intensities and transmission peak wavelengths plotted as a function of the number of grooves of the plasmonic color filter with *p* = 600 nm. The red solid line shows the fitted curve using the smoothing spline. The peak transmission wavelength was observed constantly at a wavelength of approximately 700 nm in the structures with annular grooves. Wavelength selectivity was obtained for *N* = 1 but not *N* = 0. Therefore, a color filter with a higher aperture ratio can be designed for the size of the photodiode as an array of apertures from *N* = 1. With an increasing number of grooves, the transmission peak intensity increases and saturates from *N* = 3. This means that the effective plasmonic concentration area by surface plasmon oscillation is within *N* = 3. In this study, as a prototype, we fabricated square arrays of filters with *N* = 3 to ensure the best performance of transmission efficiency.

### 5.3. Transmission Color Purity of Aperture-Array Filter with Square Configuration

**Figure 7:**
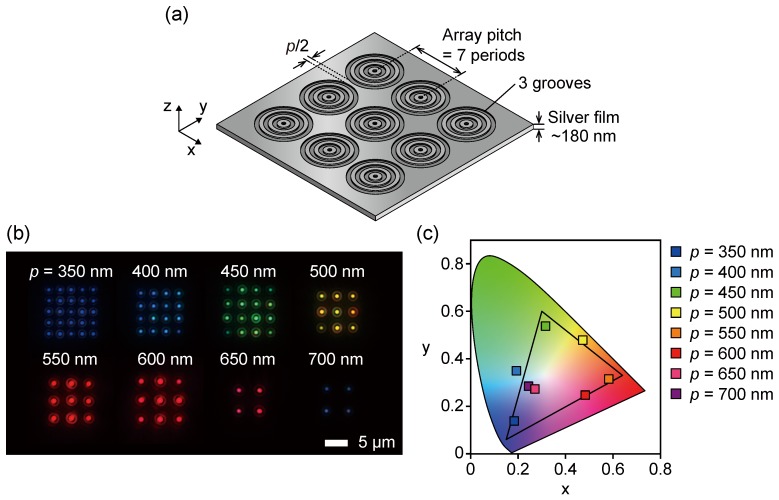
(**a**) A schematic of a 10 μm × 10 μm plasmonic color filter array with groove number *N* = 3. (**b**) Darkfield micrographs of fabricated plasmonic color filter arrays for *p* = 350–700 nm. (**c**) CIE1931 XY chromaticity diagram of the measured transmission spectra of the array.

To achieve efficient color filtering for pixel imaging, apertures with a periodic concentric corrugation were arrayed as illustrated in Figure 7a. The multi-aperture filter was fabricated as a square array of apertures, each with three circular grooves (*N* = 3). The array pitch between the subwavelength apertures is seven corrugation periods. A one pixel filter is approximately 10 μm × 10 μm and can be adjusted by the number of apertures in the array. Figure 7b shows darkfield micrographs of the transmitted light distribution of the aperture-array filter with corrugation periods ranging from 350–700 nm fabricated on a single glass substrate. The film thickness and groove depth are set to the fixed values of 180 nm and 90 nm, respectively, that are optimized for the corrugation period of 600 nm. Color-selected transmitted light corresponding to the corrugation period was clearly observed from each aperture. As shown in Figure 7b, the transmitted light with the complementary colors of CMY and the primary color of RGB was clearly observed. The optical image with the highest transmitted light intensity was obtained at the structure with a corrugation period of 600 nm, while the light intensity became lower for the short corrugation period. The reason is that the excitation efficiency depends not only on the corrugation period but also on the structural parameters of film thickness and groove depth. It is possible to improve the transmitted light intensity (specifically, the scale of the structural parameters trends to smaller for the shorter wavelength) by the optimization of the film thickness and groove depth for each target wavelength. On the other hand, in a longer corrugation period than 600 nm, the transmitted light is observed in the NIR region. The transmitted light of the NIR region was captured as an optical image with a low intensity due to an insufficient sensitivity for a color CCD camera. Figure 7c shows the CIE XY chromaticity diagram of the measured transmission spectra for the aperture-array filters. The chromaticity distribution of the aperture-array filter indicated a high color reproducibility and was very similar to that of the single aperture filter shown in Figure 5b. As with the single aperture filter, the corrugation periods corresponding to R, G, and B were *p* = 550 nm, 450 nm, and 350 nm, respectively. The XY color coordinates of the transmitted light for the RGB primary colors were almost coincident with those of the sRGB, and the transmitted light with CMY complementary colors was indicated at color coordinates equal to or exceeding the sRGB gamut, as was the case for the coordinates of the single aperture filter. The demonstration of the high color reproducibility of the aperture-array filter suggests that the filter can be designed to an appropriate size for the pixel size by adjusting the number of apertures and the number of grooves.

## 6. Conclusions

We demonstrated a high color purity plasmonic color filtering by an array of concentric corrugated metallic thin films that exhibited peak transmission wavelengths comparable to sRGB. The plasmonic color filters are fabricated by EBL and FIB techniques. The transmission characteristics of the fabricated plasmonic color filters with corrugation periods of 300–700 nm exhibited a linear peak wavelength shift from 420–700 nm with an increasing period. In the AOI dependence simulation, it was found that the applicable half cone angle was 10°. The larger AOI transmitted longer wavelength bands with a lower transmission efficiency due to the surface plasmon dispersion. Since our plasmonic color filter demonstrated a higher color purity than previously reported plasmonic color filters such as nanohole arrays, the transmitted light exhibited a multiband color in both the sRGB and CMY complementary color gamuts. In addition, NIR light was transmitted by the filter with larger corrugation periods. This high color purity of multiband transmission from the visible to the NIR is of interest in bio-imaging, day/night vision cameras, remote sensing, etc.

## References

[1] Tominaga S. (1996). Multichannel vision system for estimating surface and illumination functions. J. Opt. Soc. Am. A.

[2] Burns P.D., Berns R.S. Analysis multispectral image capture. http://losburns.com/imaging/pbpubs/18cic1996burnsberns.pdf.

[3] Fukuda H., Uchiyama T., Haneishi H., Yamaguchi M., Ohyama N. Development of a 16-band multispectral image archiving system. Proceedings of the Electronic Imaging 2005.

[4] Gat N. Imaging spectroscopy using tunable filters: A review. Proceedings of the AeroSense 2000.

[5] Tominaga S. (1999). Spectral imaging by a multichannel camera. J. Electr. Imaging.

[6] Brauers J., Aach T. A color filter array based multispectral camera. Proceedings of the 12th Workshop Farbbildverarbeitung.

[7] Li Y., Majumder A., Zhang H., Gopi M. (2018). Optimized multi-spectral filter array based imaging of natural scenes. Sensors.

[8] Yasuma F., Mitsunaga T., Iso D., Nayar S.K. (2010). Generalized assorted pixel camera: Postcapture control of resolution, dynamic range, and spectrum. IEEE Trans. Image Process..

[9] Bayer B. (1976). Color Imaging Array. U.S. Patent.

[10] Degiron A., Ebbesen T.W. (2005). The role of localized surface plasmon modes in the enhanced transmission of periodic subwavelength apertures. J. Opt. A Pure Appl. Opt..

[11] Laux E., Genet C., Skauli T., Ebbesen T.W. (2008). Plasmonic photon sorters for spectral and polarimetric imaging. Nat. Photonics.

[12] Inoue D., Miura A., Nomura T., Fujikawa H., Sato K., Ikeda N., Tsuya D., Sugimoto Y., Koide Y. (2011). Polarization independent visible color filter comprising an aluminum film with surface-plasmon enhanced transmission through a subwavelength array of holes. Appl. Phys. Lett..

[13] Chen Q., Das D., Chitnis D., Walls K., Drysdale T.D., Collins S., Cumming D.R.S. (2012). A CMOS image sensor integrated with plasmonic colour filters. Plasmonics.

[14] Yokogawa S., Burgos S.P., Atwater H.A. (2012). Plasmonic color filters for CMOS image sensor applications. Nano Lett..

[15] Burgos S.P., Yokogawa S., Atwater H.A. (2013). Color imaging via nearest neighbor hole coupling in plasmonic color filters integrated onto a complementary metal-oxide semiconductor image sensor. ACS Nano.

[16] Zeng B., Gao Y., Bartoli F.J. (2013). Ultrathin nanostructured metals for highly transmissive plasmonic subtractive color filters. Sci. Rep..

[17] Fleischman D., Sweatlock L.A., Murakami H., Atwater H. (2017). Hyper-selective plasmonic color filters. Opt. Express.

[18] Heydari E., Sperling J.R., Neale S.L., Clark A.W. (2017). Plasmonic color filters as dual-state nanopixels for high-density microimage encoding. Adv. Funct. Mater..

[19] Chen Q., Hu X., Wen L., Yu Y., Cumming D.R. (2016). Nanophotonic image sensors. Small.

[20] Ebbesen T.W., Lezec H.J., Ghaemi H.F., Thio T., Wolff P.A. (1998). Extraordinary optical transmission through sub-wavelength hole arrays. Nature.

[21] Miyamichi A., Ono A., Kamehama H., Kagawa K., Yasutomi K., Kawahito S. (2018). Multi-band plasmonic color filters for visible-to-near-infrared image sensors. Opt. Express.

[22] Lezec H.J., Degiron A., Devaux E., Linke R.A., Martin-Moreno L., Garcia-Vidal F.J., Ebbesen T.W. (2002). Beaming light from a subwavelength aperture. Science.

[23] Ishi T., Fujikata J., Makita K., Baba T., Ohashi K. (2002). Si nano-photodiode with a surface plasmon antenna. Jpn. J. Appl. Phys..

[24] Chen Z., Wang X., Liang R. (2014). RGB-NIR multispectral camera. Opt. Express.

[25] Tang H., Zhang X., Zhuo S., Chen F., Kutulakos K.N., Shen L. High resolution photography with an RGB-infrared camera. Proceedings of the 2015 IEEE International Conference on Computational Photography (ICCP).

[26] Teranaka H., Monno Y., Tanaka M., Okutomi M. Single-sensor RGB and NIR image acquisition: Toward optimal performance by taking account of CFA pattern, demosaicking, and color correction. Proceedings of the IS&T Electronic Imaging 2016 (EI2016).

